# Changes in socioeconomic resources and mental health after the second COVID-19 wave (2020–2021): a longitudinal study in Switzerland

**DOI:** 10.1186/s12939-023-01853-2

**Published:** 2023-03-23

**Authors:** Stefano Tancredi, Agnė Ulytė, Cornelia Wagner, Dirk Keidel, Melissa Witzig, Medea Imboden, Nicole Probst-Hensch, Rebecca Amati, Emiliano Albanese, Sara Levati, Luca Crivelli, Philipp Kohler, Alexia Cusini, Christian Kahlert, Erika Harju, Gisela Michel, Chantal Lüdi, Natalia Ortega, Stéphanie Baggio, Patricia Chocano-Bedoya, Nicolas Rodondi, Tala Ballouz, Anja Frei, Marco Kaufmann, Viktor Von Wyl, Elsa Lorthe, Hélène Baysson, Silvia Stringhini, Valentine Schneider, Laurent Kaufmann, Frank Wieber, Thomas Volken, Annina Zysset, Julia Dratva, Stéphane Cullati, the Corona Immunitas Research Group, Antonio Amendola, Alexia Anagnostopoulos, Daniela Anker, Anna Maria Annoni, Hélène Aschmann, Andrew Azman, Antoine Bal, Kleona Bezani, Annette Blattmann, Patrick Bleich, Murielle Bochud, Patrick Bodenmann, Gaëlle Bryand Rumley, Peter Buttaroni, Audrey Butty, Anne Linda Camerini, Arnaud Chiolero, Patricia Orializ Chocano-Bedoya, Prune Collombet, Laurie Corna, Valérie D’Acremont, Diana Sofia Da Costa Santos, Agathe Deschamps, Anja Domenghino, Richard Dubos, Roxane Dumont, Olivier Duperrex, Julien Dupraz, Malik Egger, Emna El-May, Nacira El Merjani, Nathalie Engler, Adina Mihaela Epure, Lukas Erksam, Sandrine Estoppey, Marta Fadda, Vincent Faivre, Jan Fehr, Andrea Felappi, Maddalena Fiordelli, Antoine Flahault, Luc Fornerod, Cristina Fragoso Corti, Natalie Francioli, Marion Frangville, Irène Frank, Giovanni Franscella, Marco Geigges, Semira Gonseth Nusslé, Clément Graindorge, Idris Guessous, Séverine Harnal, Emilie Jendly, Ayoung Jeong, Christian R Kahlert, Laurent Kaiser, Simone Kessler, Christine Krähenbühl, Susi Kriemler, Julien Lamour, Pierre Lescuyer, Andrea Loizeau, Chantal Luedi, Jean-Luc Magnin, Chantal Martinez, Eric Masserey, Dominik Menges, Rosalba Morese, Nicolai Mösli, Natacha Noël, Daniel Henry Paris, Jérôme Pasquier, Francesco Pennacchio, Stefan Pfister, Giovanni Piumatti, Géraldine Poulain, Caroline Pugin, Milo Puhan, Nick Pullen, Thomas Radtke, Manuela Rasi, Aude Richard, Viviane Richard, Claude-François Robert, Pierre-Yves Rodondi, Serena Sabatini, Khadija Samir, Javier Sanchis Zozaya, Virginie Schlüter, Alexia Schmid, Maria Schüpbach, Nathalie Schwab, Claire Semaani, Alexandre Speierer, Amélie Steiner-Dubuis, Stéphanie Testini, Julien Thabard, Mauro Tonolla, Nicolas Troillet, Agne Ulyte, Sophie Vassaux, Thomas Vermes, Jennifer Villers, Rylana Wenger, Erin West, Ania Wisniak, María-Eugenia Zaballa, Kyra Zens, Claire Zuppinger

**Affiliations:** 1 Population Health Laboratory (#PopHealthLab), University of Fribourg, Fribourg, Switzerland; 2 Biostatistics and Prevention Institute, University of Zurich, Zurich, Switzerland; 3 Swiss Tropical and Public Health Institute, Allschwil, Switzerland; 4 University of Basel, Basel, Switzerland; 5 Institute of Public Health, Faculty of Biomedical Sciences, Università della Svizzera Italiana, Lugano, Switzerland; 6 Department of Business Economics, Health and Social Care at the University of Applied Sciences and Arts of Southern Switzerland, Manno, Switzerland; 7 Division of Infectious Diseases and Hospital Epidemiology, Kantonsspital St Gallen, St-Gallen, Switzerland; 8 Department of Infectious Diseases and Hospital Epidemiology, Children’s Hospital of Eastern Switzerland, St Gallen, Switzerland; 9 Department Health Sciences and Medicine, University of Lucerne, Lucerne, Switzerland; 10 Institute of Primary Health Care (BIHAM), University of Bern, Bern, Switzerland; 11 Division of Prison Health, Geneva University Hospitals & University of Geneva, Geneva, Switzerland; 12 Department of General Internal Medicine, Inselspital, Bern University Hospital, University of Bern, Bern, Switzerland; 13 Epidemiology, Biostatistics and Prevention Institute, University of Zurich, Zurich, Switzerland; 14 Unit of Population Epidemiology, Division of Primary Care Medicine, Geneva University Hospitals, Geneva, Switzerland; 15 Department of Health and Community Medicine, Faculty of Medicine, University of Geneva, Geneva, Switzerland; 16 Cantonal Public Health Service of the Canton of Neuchâtel, Neuchâtel, Switzerland; 17 Zurich University of Applied Sciences, Institute of Public Health, Winterthur, Switzerland; 18 Department of Psychology, University of Konstanz, Konstanz, Germany; 19 Department of Readaptation and Geriatrics, University of Geneva, Geneva, Switzerland

**Keywords:** COVID-19, Depressive symptoms, Anxiety, Stress, Socioeconomic condition, Financial resources

## Abstract

**Background:**

During the 2020/2021 winter, the labour market was under the impact of the COVID-19 pandemic. Changes in socioeconomic resources during this period could have influenced individual mental health. This association may have been mitigated or exacerbated by subjective risk perceptions, such as perceived risk of getting infected with SARS-CoV-2 or perception of the national economic situation. Therefore, we aimed to determine if changes in financial resources and employment situation during and after the second COVID-19 wave were prospectively associated with depression, anxiety and stress, and whether perceptions of the national economic situation and of the risk of getting infected modified this association.

**Methods:**

One thousand seven hundred fifty nine participants from a nation-wide population-based eCohort in Switzerland were followed between November 2020 and September 2021. Financial resources and employment status were assessed twice (Nov2020–Mar2021, May–Jul 2021). Mental health was assessed after the second measurement of financial resources and employment status, using the Depression, Anxiety and Stress Scale (DASS-21). We modelled DASS-21 scores with linear regression, adjusting for demographics, health status, social relationships and changes in workload, and tested interactions with subjective risk perceptions.

**Results:**

We observed scores above thresholds for normal levels for 16% (95%CI = 15–18) of participants for depression, 8% (95%CI = 7–10) for anxiety, and 10% (95%CI = 9–12) for stress. Compared to continuously comfortable or sufficient financial resources, continuously precarious or insufficient resources were associated with worse scores for all outcomes. Increased financial resources were associated with higher anxiety. In the working-age group, shifting from full to part-time employment was associated with higher stress and anxiety. Perceiving the Swiss economic situation as worrisome was associated with higher anxiety in participants who lost financial resources or had continuously precarious or insufficient resources.

**Conclusion:**

This study confirms the association of economic stressors and mental health during the COVID-19 pandemic and highlights the exacerbating role of subjective risk perception on this association.

**Supplementary Information:**

The online version contains supplementary material available at 10.1186/s12939-023-01853-2.

## Introduction

The coronavirus disease 2019 (COVID-19) pandemic prompted nationwide lockdowns and restrictive measures around the world, leading to profound effects on the economy and on the labour market. The pandemic slowed down economic activities and led to a rise in the unemployment rate in multiple countries, with millions of people losing their jobs [1]. During the winter of 2020–2021, Switzerland was experiencing the second wave of the pandemic. During and after it, the country implemented lighter mitigation strategies compared to other European countries [2]. Nevertheless, Swiss labour market suffered from the economic consequences caused by COVID-19. Families’ private debts increased due to the pandemic [3] and the unemployment rate in Switzerland, as defined by the International Labour Organisation, increased both in the last quarter of 2020 and at the beginning of 2021 [4, 5]. Although the mean disposable income of Swiss households remained stable in 2020 (compared to 2019) [6], 11.3% of the general population experienced a loss of income due to the pandemic in 2021 [7].

The loss of socioeconomic resources may have impacted mental health and mental well-being. Previous studies have shown an association between financial hardship and poorer mental health [8–15]. Losing financial resources increases psychosocial stress and can lead to a loss of flexible resources, such as power or prestige, that can be used to minimize the consequences of a stressful event [16, 17]. Moreover, the pandemic may have exacerbated economic inequalities; existing evidence suggests that the burden of the pandemic is not equally distributed in the population, with a higher burden and worse mental health in people with lower socioeconomic status [18–20]. Further, the population’s perception of the national economic situation and the individual perception of the risk of getting infected with COVID-19 could have also influenced mental health. Previous studies have shown a link between a higher risk perception of getting infected and worse mental health [21–23], probably due to the fear of falling ill, losing a loved one, and of possible social or economic consequences of isolation. Besides their direct individual impact on mental health, these factors can modify the association between changes in socioeconomic resources and mental health, exacerbating the detrimental effects of the loss of socioeconomic resources on mental health. To our knowledge, this has not been reported yet in any study.

Therefore, in this study, we aimed to determine [a] if changes in financial resources and employment situation during and after the second COVID-19 wave in 2020–2021 were prospectively associated with self-reported depression, anxiety and stress symptoms, and [b] whether perceptions of the national economic situation and of the risk of getting infected modified this association. Additionally, as the risk of financial loss was higher among people in the working age, we also investigated these associations in the sub-group of not retired persons.

## Methods

### Study population and design

The Corona Immunitas digital follow-up (CI-DFU) eCohort is a population-based digital longitudinal study [24]. The cohort is part of the Corona Immunitas research programme [25], a nation-wide seroprevalence study coordinated by the Swiss School of Public Health (SSPH +), based on randomly selected adults living in Switzerland. Participants of Corona Immunitas were invited to join the CI-DFU eCohort if they were at least 20 years old and had a valid email address and internet access. Participants could answer the CI-DFU questionnaires by choosing between four different languages: German, French, Italian and English. The questionnaires were completed online (data were collected using REDCap, Research Electronic Data Capture). Weekly participation rates ranged from 75% to 88.6% [24].

The present study included 1′759 participants from the CI-DFU eCohort, living in several cantons in Switzerland (Bern, Fribourg, Neuchatel, St-Gallen, Ticino, Winterthur and Zurich). We administered a questionnaire to assess the financial resources and employment situation of participants twice: first, between November 2020 and March 2021 (hereafter: first questionnaire = Q1) and second, between May and July 2021 (hereafter: second questionnaire = Q2). We assessed mental health using the Depression, Anxiety and Stress Scale (DASS-21) between May and September 2021. Figure S1 (Additional file 1: Figure S1) shows a timeline of the questionnaires’ administration together with the COVID-19 pandemic context. We included participants who [1] filled the questionnaire on financial resources and employment situation both times, with at least 60 days between the first (Q1) and the second (Q2) questionnaire’s response, and [2] completed the mental health questionnaire at least 7 days after Q2 (May – September 2021; median 28 days after the second questionnaire, range 7 to 116 days). We excluded participants with missing data on the outcome variables or on any other analyzed covariates (a comparison between the characteristics of the included and excluded population is reported in additional file 1, table S1 and S2; no differences were found between the excluded and included population according to working status). Figure S2 (Additional file 1: Figure S2) shows a flow diagram of study participants. The reporting of this study followed the “Strengthening the reporting of observational studies in epidemiology” (STROBE) statement [26].

### Outcome measures

We measured mental health using the Depression, Anxiety and Stress Scale (DASS-21) [27]. The DASS-21 is a 21-item self-administered questionnaire developed to screen and assess mental health symptoms and their severity, and comprises three 7-item subscales for depression (Cronbach alpha 0.93 in our study population), anxiety (Cronbach alpha 0.85) and stress symptoms (Cronbach alpha 0.92). The principal component analysis confirmed the one-dimensionality of each subscale in this study population. DASS-21 is commonly used in health research to assess symptoms of depression, anxiety and stress in the general population and in clinical settings [28]. We used the validated translations of the German [29], French [30] and Italian [31] versions of the DASS-21. Respondents reported the frequency of their depression, anxiety, and stress symptoms in the last seven days on a four-point Likert scale (never; sometimes; often; and almost always). Continuous scores for depression, anxiety and stress-related symptoms were computed following the usual standard [32], where each category score is calculated by summing the subscale item scores and multiplying by two. The final scores range from 0 (no symptoms) to 42 (worst symptoms) for each subscale, and can be categorized to severity levels according to the standard guidance (a score is considered “normal” if it is below 9 for depression, 7 for anxiety and 14 for stress) [32]. DASS-21 severity categories are described in supplementary table S3 (Additional file 1: Table S3).

### Main predictors

We administered a structured questionnaire to assess the financial resources and employment situation of participants twice (between November 2020 and March 2021 (Q1) and between May and July 2021 (Q2)). Participants were asked to assess their financial resources with the prompt “In the past 6 months, would you say that financially…” followed by response options “You are comfortable, money is not a concern and it is easy to save money” (hereafter, comfortable), “Your income allows you to cover your expenses and to compensate for any minor contingencies” (hereafter, sufficient), “You need to be careful with your expenses and an unforeseen event could put you in financial difficulty” (hereafter, precarious), “You are unable to cover your needs with your income and need external support to function (debt, credit, various financial aids)” (hereafter, insufficient). We assessed the following combinations of changes in financial resources: comfortable/sufficient resources at Q1 and Q2 (used as reference category in our analyses); comfortable/sufficient resources at Q1 and lower at Q2; precarious/insufficient resources at Q1 and higher at Q2; precarious/insufficient resources both at Q1 and Q2.

We categorised employment status according to the participants’ situation during the month prior to the completion of the questionnaire into the following groups: employed full-time (minimum 37 h weekly), employed part-time, self-employed, and other employment positions (unemployed, retired, student, at home (domestic work, children care) or in another situation). All possible combinations between these groups were assessed (i.e., Full-time or part-time at both Q1 and Q2; full-time at Q1 and part-time at Q2; part-time at Q1 and full-time at Q2; full- or part-time at Q1 and self-employed at Q2; self-employed at Q1 and full- or part-time at Q2; Self-employed at Q1 and Q2; full- or part-time at Q1 and other at Q2; other employment at Q1 and Q2; other at Q1 and full- or part-time at Q2). Employment was further grouped as a binary variable of retired versus not retired (all other employment options).

### Potential moderators

Worries on the economic situation in Switzerland were assessed on a Likert scale from 1 (not worried at all) to 5 (extremely worried) in response to the question “How worried are you about the current coronavirus situation in the following area: the general economic situation in Switzerland”. The risk of getting infected in the last week was reported as a response to the statement “In the last 7 days, do you think the risk of being infected with coronavirus (SARS-CoV-2) is…” on a sliding scale from 0 (no risk) to 100 (very high risk), divided by 10. We reported median values assessed in weekly (risk of infection) and monthly (economic situation) questionnaires between Q1 and Q2. In a sensitivity analysis, we used the median reported values for each month between January and April 2021.

### Control variables

We collected information on the demographic characteristics, health status and social relationships of the participants. For demographics, we assessed participants’ age (continuous), sex (women, men, other), and language preference for filling the questionnaires (largely corresponding to the linguistic regions in Switzerland). Language preference was not recorded for some participants from the cantons of Zurich and Ticino, and instead the dominant language of the canton was recorded (German and Italian, respectively). Health status included the number of chronic conditions, having reported a positive SARS-CoV-2 test (PCR or antigen test) by the time of completing the Q2 questionnaire and vaccination status by the time of completing the DASS-21 questionnaire. Chronic conditions were assessed with the question “Do you suffer from one or more of the following diseases? cancer, immunological diseases, cardiovascular diseases, diabetes, hypertension and respiratory diseases”. Social relationships assessed whether the participant was living alone in the household and their reported loneliness (median of Three-Item Loneliness Scale reported between Q1 and Q2, range from 3 (lowest) to 15 (highest) loneliness) [33]. Change in workload (no change, increase, decrease) in the past 6 months was also included as a control variable as measured in Q1 questionnaire.

### Statistical analysis

We presented descriptive statistics of the cohort, both for the total sample and stratified by being in the working-age population or retired. We modelled depression, anxiety and stress outcome scores in univariable and multivariable linear regressions. The main predictors in the multivariable models were financial resources and employment status at Q1, and their changes by Q2. The models also adjusted for demographic characteristics (age, sex, language), health status (chronic conditions, positive COVID-19 test result, being vaccinated against SARS-CoV-2), social relationships (loneliness, living alone) and changes in workload. We tested the effect of an interaction between changes in the socioeconomic resources (financial resources and employment situation) and potential moderators (perceived Swiss economic situation and the perceived risk of getting infected with SARS-CoV-2) by adding each potential moderator in a separate multivariable linear regression model. We ran multivariable models for the entire sample and separately for the working-age population and retired persons. A theoretical framework of the study is shown in supplemental figure S3 (Additional file 1: Figure S3).

We also performed several sensitivity analyses: [1] we ran logistic regression models for the full study population with dichotomized mental health outcomes (normal or mild vs moderate, severe or extremely severe categories). We re-ran [2] the linear models using a subset of the full study population including monthly rather than median values of risk perceptions, to account for potential influence of changing risk perceptions over time. We re-ran [3] the linear multivariate models using multiple imputation by chained equation to account for missing information. Statistical analyses were performed using R statistical software (version 4.0.2, R Foundation for Statistical Computing, Vienna, Austria; packages used for statistics and tables: stats 4.0.2, gtsummary 1.4.1, mice 3.14.0; packages used for visualizations and plots: ggplot2 3.3.3, sjPlot 2.8.9).

## Results

### Respondents’ characteristics

We included 1′759 participants (79% of the included participants filled the Q1 questionnaire between November and December 2020; 99% filled the Q2 questionnaire in May 2021; and 98% of participants filled the DASS-21 questionnaire between June and July 2021). The majority of participants (79%) filled out the DASS questionnaire 28 days or more after Q2. Descriptive characteristics of the participants are presented in Table 1. The median age was 53 years (IQR = 40–64) and 52% of participants were female. Mental health scores above thresholds for normal levels were reported by 16% (95%CI = 15–18) of participants for depression, 8% (95%CI = 7–10) for anxiety, and 10% (95%CI = 9–12) for stress. The median reported scores for depression, anxiety and stress were 0 (IQR = 0–6), 0 (IQR = 0–2) and 2 (IQR = 0–10), respectively; median scores for worries on the economic situation in Switzerland and risk of getting infected were 3 (out of 5, IQR = 2–4) and 4.5 (out of 10, IQR = 2.1–6.5), respectively. Differences and similarities between retired and not retired participants are reported in Table 1. The proportion of scores above the threshold for normal level for the three mental health outcomes was lower among retired participants compared to non-retired participants (depression: 12% vs 18%, *p* = 0.002; anxiety: 6% vs 9%, *p* = 0.017; stress: 5% vs 12%, *p* < 0.001).

**Table 1:** Descriptive characteristics of the studied population

	**Overall**	**Not retired**^**a**^	**Retired**^**a**^
**N**	1759	1336	423
**Age,** median (IQR)	53 (40—64)	48 (36—56)	70 (67—74)
**Sex**			
Female	909 (52%)	722 (54%)	187 (44%)
Male	846 (48%)	610 (46%)	236 (56%)
Other	4(0.1%)	4 (0.3%)	0 (0%)
**One or more chronic health conditions**	621 (35%)	356 (27%)	265 (63%)
**SARS-CoV-2 positive test**	154 (8.8%)	130 (9.7%)	24 (5.7%)
**SARS-CoV-2 Vaccinated**	397 (23%)	117 (8.8%)	280 (66%)
**Language**			
German	886 (50%)	631(47%)	255 (60%)
Italian	505 (29%)	481 (36%)	24 (5.7%)
French	353 (20%)	211 (16%)	142 (34%)
English	15 (0.9%)	13 (1.0%)	2 (0.5%)
**Living alone**	278 (16%)	190 (14%)	88 (21%)
**Loneliness score,** median (IQR)^b^	5 (4—7)	5 (3—7)	6 (4—8)
**Worries about Swiss economy,** median (IQR)^b^	3 (2—4)	3 (2—4)	3 (3—4)
**Perceived risk to be infected,** median (IQR)^b^	4.4 (2.1—6.5)	4.4 (2.1 – 6.4)	4.6 (1.9 – 6.9)
**Change in workload**			
No change	1,046 (78%)	1,046 (78%)	0 (0%)
Increase	150 (11%)	150 (11%)	0 (0%)
Decrease	140 (10%)	140 (10%)	0 (0%)
**Mental health outcomes**^c^			
**Depression**			
Median (IQR)	0 (0—6)	0 (0—6)	0 (0—4)
Normal	1,472 (84%)	1,102 (82%)	370 (87%)
Mild to moderate	227 (13%)	183 (14%)	44 (10%)
Sever to extremely severe	60 (3.4%)	51 (3.8%)	9 (2.1%)
**Anxiety**			
Median (IQR)	0 (0—2)	0 (0—2)	0 (0—2)
Normal	1,613 (92%)	1,214 (91%)	399 (94%)
Mild to moderate	107 (6.1%)	87 (6.6%)	20 (4.7%)
Sever to extremely severe	39 (2.2%)	35 (2.6%)	4 (0.9%)
**Stress**			
Median (IQR)	2 (0—10)	2 (0—10)	0 (0—6)
Normal	1,581 (90%)	1,178 (88%)	403 (95%)
Mild to moderate	136 (7.7%)	120 (9.0%)	16 (3.8%)
Sever to extremely severe	42 (2.4%)	38 (2.8%)	4 (0.9%)

a: Participants were grouped in retired or not retired categories according to their employment situation at Q1

b: Loneliness score ranged from 3 (smallest) to 15 (highest). Worries about Swiss economic situation ranged between 1 (smallest) to 5 (highest). Perceived risk to be infected ranged between 0 (no risk) to 10 (very high)

c: Mental health outcomes were assessed between May and September 2021 using the DASS-21 score, with minimum 0 (best outcome) and maximum 42 (worst outcome) score. See details on the instruments in Methods

The cross-sectional distribution of the reported financial resources was similar in Q1 and Q2 among the retired and not retired participants (Table 2). Not retired participants reported insufficient or precarious financial resources more often than retired participants both at Q1 (17% vs 9%) and Q2 (16% vs 9%). Distributions of employment type were also similar among the not retired participants in Q1 and Q2. Proportion of changes in financial resources and employment situation are reported in table S4 (Additional file 1: Table S4).

**Table 2:** Financial resources and employment situation of the participants

	**Q1**	**Q2**	***P* values **^**a**^
***Overall (n* = *1759)***			
**Financial resources**			0.6
Comfortable	753 (43%)	783 (45%)	
Sufficient	736 (42%)	724 (41%)	
Precarious	241 (14%)	219 (12%)	
Insufficient	29 (1.6%)	33 (1.9%)	
***Retired (n* = *404)***			
**Financial resources**			> 0.9
Comfortable	175 (43%)	176 (44%)	
Sufficient	191 (47%)	190 (47%)	
Precarious	37 (9.2%)	38 (9.4%)	
Insufficient	1 (0.2%)	0 (0%)	
***Not retired (n* = *1286)***			
**Financial resources**			0.6
Comfortable	552 (43%)	577 (45%)	
Sufficient	512 (40%)	504 (39%)	
Precarious	195 (15%)	176 (14%)	
Insufficient	27 (2.1%)	29 (2.3%)	
**Employment situation**			> 0.9
Full-time	641 (50%)	635 (49%)	
Part-time	349 (27%)	354 (28%)	
Self employed	127 (9.9%)	131 (10%)	
Other ^b^	169 (13%)	166 (13%)	

Note:

Q1: first questionnaire on financial resources and employment situation; November 2020 – March 2021

Q2: second questionnaire on financial resources and employment situation; May – July 2021

Not retired n is smaller than the n reported in Table 2 because it refers to participants who were not retired both at Q1 and Q2

a: P values were computed using Pearson’s Chi-squared test or Fisher’s exact test

b: unemployed, student, at home, or in another situation

### Changes in financial resources

**Fig. 1:**
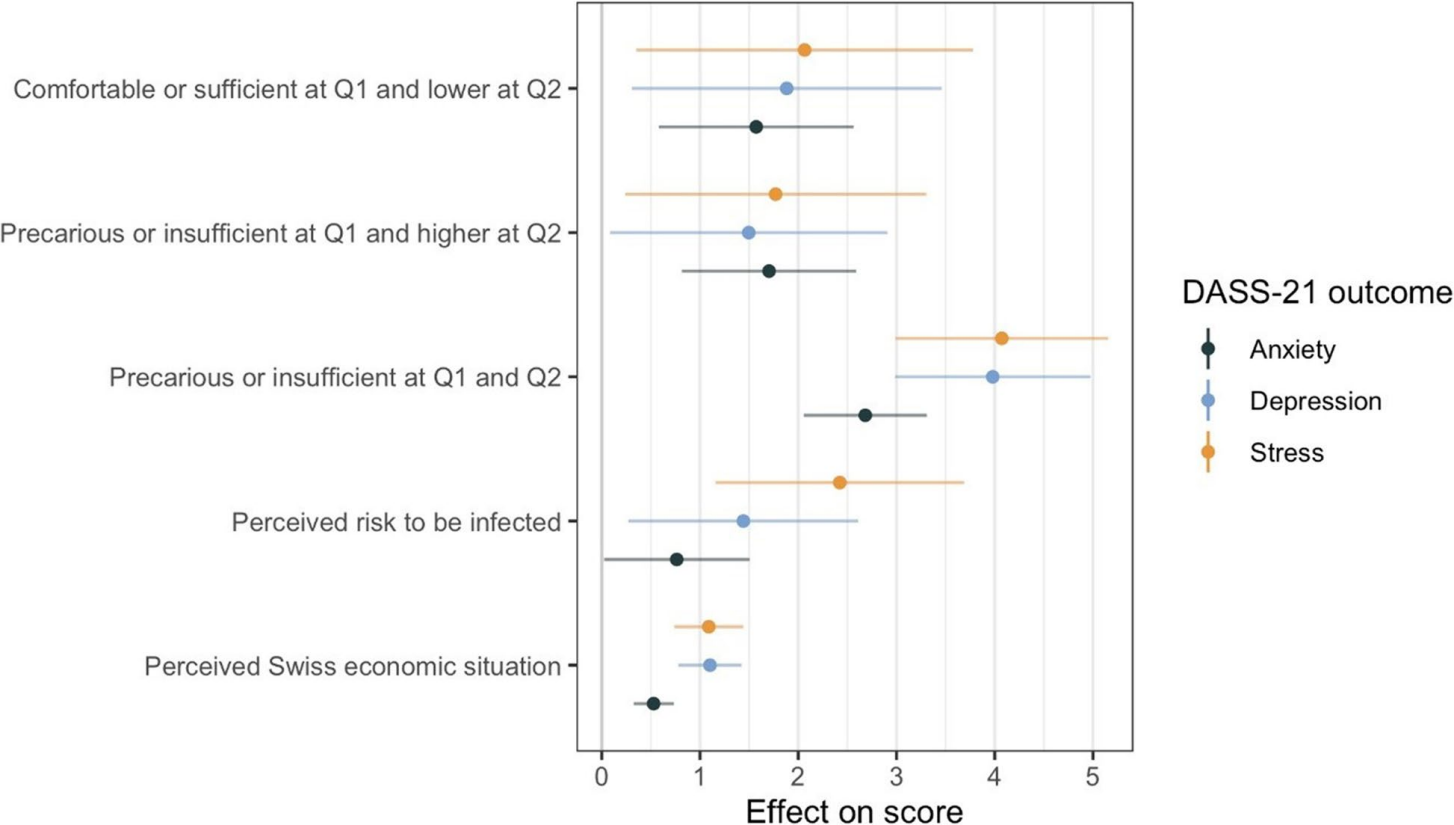
Changes in financial resources, risk and economic perception, and mental health outcomes: univariable regression. Note: a positive coefficient (effect on score) means a higher DASS-21 score (more symptoms) Q1: first questionnaire on financial resources and employment situation; November 2020 – March 2021. Q2: second questionnaire on financial resources and employment situation; May – July 2021. Financial resources categories are compared to the reference category of “comfortable or sufficient resources at both Q1 and Q2”

In univariable analyses, stable precarious or insufficient financial resources (both at Q1 and Q2) were associated with worse (higher) anxiety, depression and stress scores (Fig. 1). Participants who either improved or worsened their financial situation (precarious or insufficient resources at Q1 and higher at Q2, or comfortable or sufficient resources at Q1 and lower at Q2) also reported worse scores in all three outcomes. A higher perceived risk to be infected and a worse perception of the Swiss economic situation were associated with worse scores in all outcomes (Fig. 1).

**Fig. 2:**
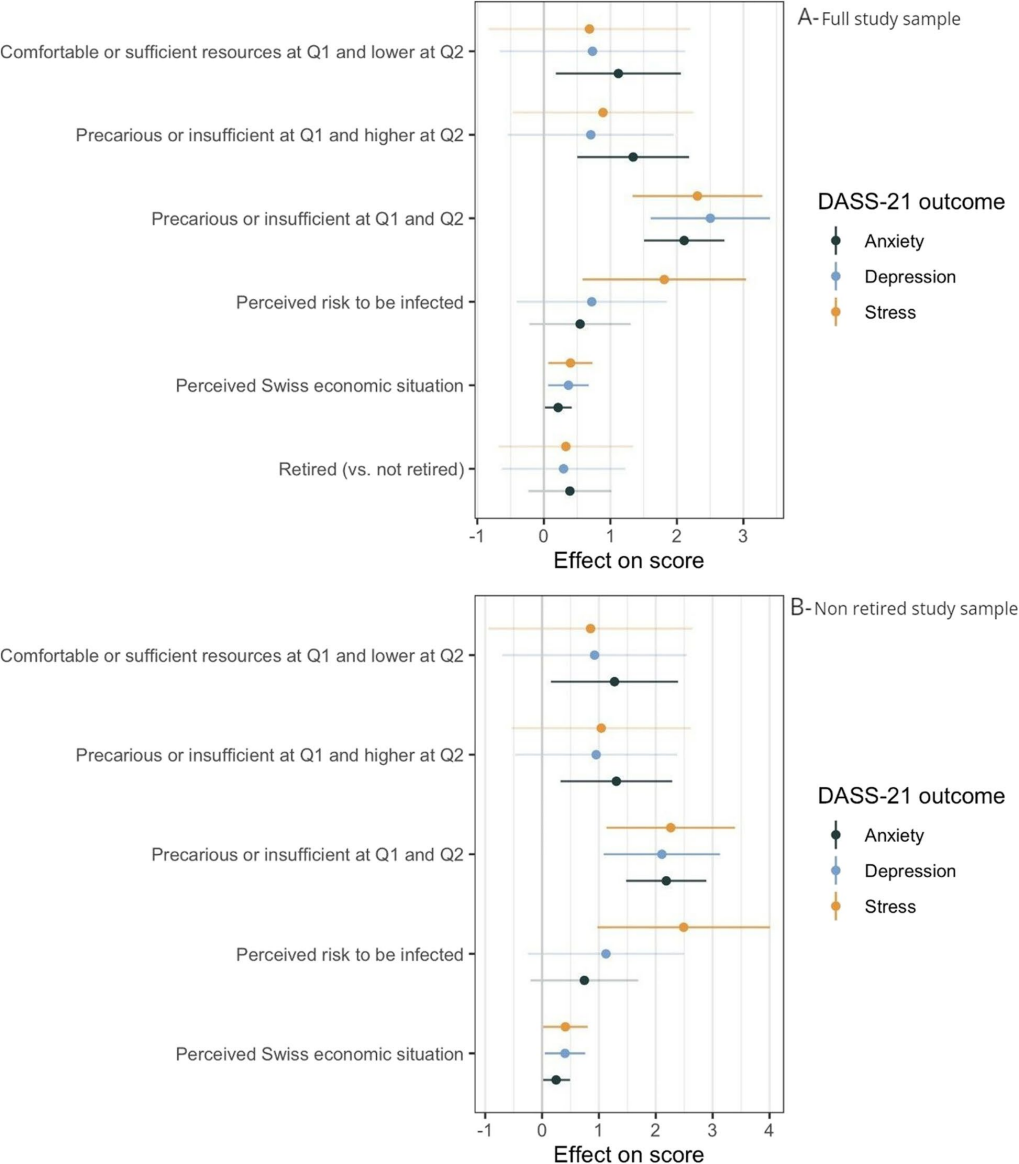
Changes in financial resources, perceived risk of infection and economic perceptions, and mental health outcomes: multivariable regression. Note: a positive coefficient (effect on score) means a higher DASS-21 score (more symptoms) Q1: first questionnaire on financial resources and employment situation; November 2020 – March 2021. Q2: second questionnaire on financial resources and employment situation; May – July 2021. Model estimates are adjusted for sex, age, number of chronic health conditions, positive COVID-19 test before Q2, vaccination status, size of household (living alone vs living with other persons), median loneliness score, employment status, changes in workload and language. Financial resources categories are compared to the reference category of “comfortable or sufficient resources at both Q1 and Q2”

In the multivariable model of the full study population (Fig. 2A), having precarious or insufficient resources both at Q1 and Q2 was predictive of worse scores for all outcomes (compared to respondents with comfortable resources at both time points). Both improvement and worsening of participants’ financial situation were associated with higher anxiety scores.

Higher perceived risk of getting infected was associated with higher stress scores and a worse perception of the Swiss economic situation with slightly higher stress, anxiety and depression scores. Similar associations were observed in the non-retired subset of the study population (Fig. 2B). Coefficients and confidence intervals of the multivariable model are reported in table S5 (Additional file 1: Table S5).

### Changes in employment situation

**Fig. 3:**
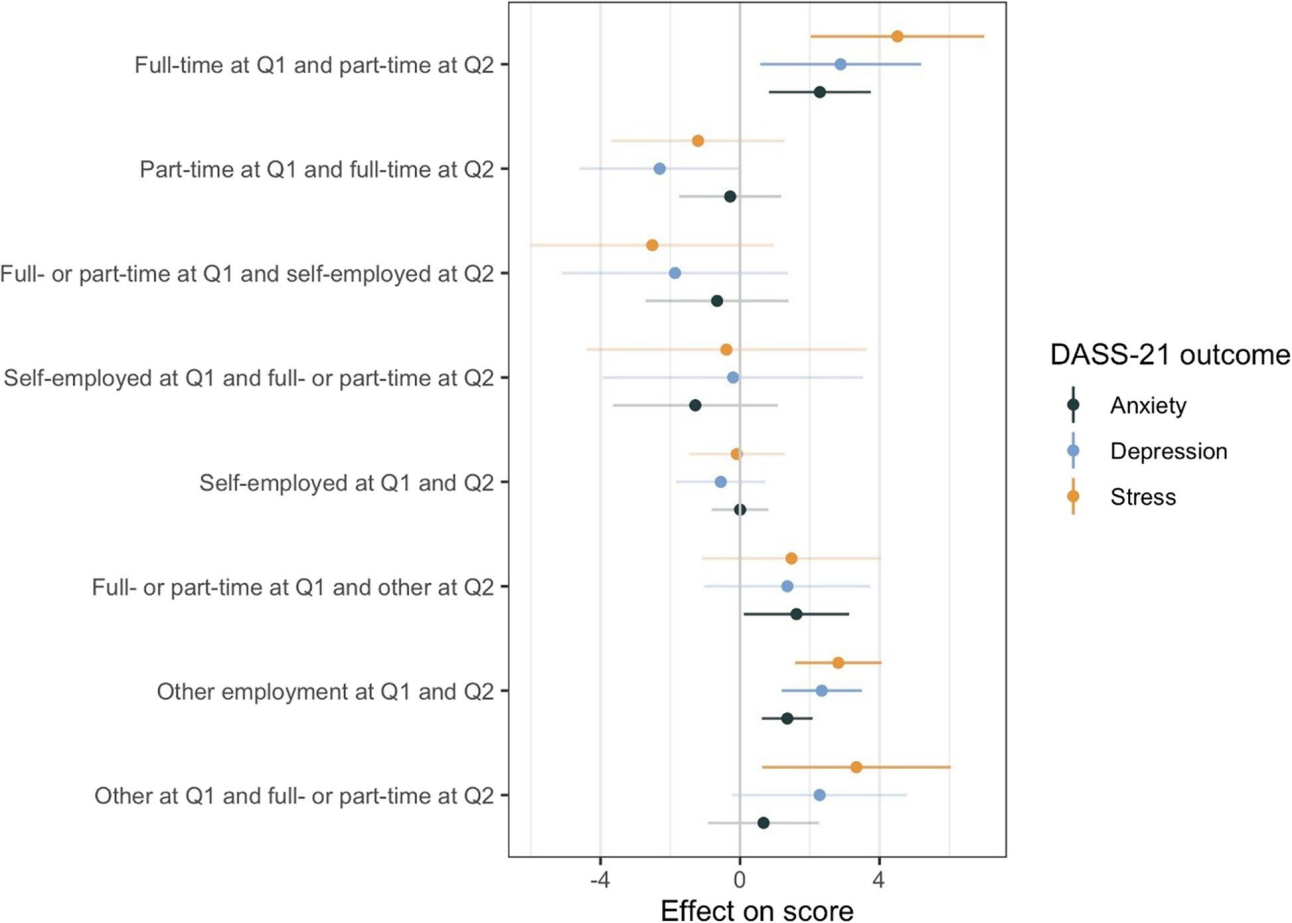
Changes in employment situation and mental health outcomes among working-age participants: univariable regression. Note: a positive coefficient (effect on score) means a higher DASS-21 score (more symptoms) Q1: first questionnaire on financial resources and employment situation; November 2020 – March 2021. Q2: second questionnaire on financial resources and employment situation; May – July 2021. Change in employment situation is compared to a reference category of “full- or part-time employed at both Q1 and Q2”

Results of the univariate analyses among working-age participants showed that changing from full to part-time employment and being in other employment positions (unemployed, students, people at home, people in another situation) than employed or self-employed both at Q1 and Q2 were associated with higher scores in all outcomes (Fig. 3). Shifting from full or part-time employment to other employment positions was associated with higher anxiety. Shifting from other employment positions to full or part-time was associated with higher stress.

**Fig. 4:**
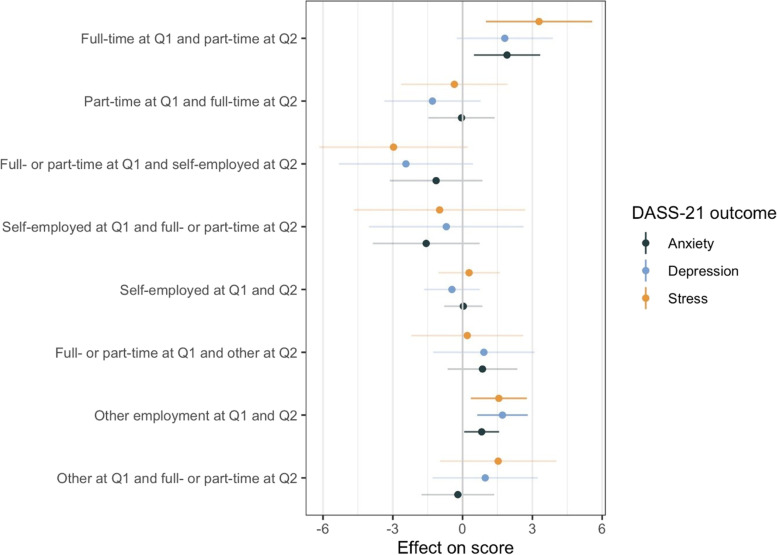
Changes in employment situation and mental health outcomes among working-age participants: multivariable regression. Note: a positive coefficient (effect on score) means a higher DASS-21 score (more symptoms). Q1: first questionnaire on financial resources and employment situation; November 2020 – March 2021. Q2: second questionnaire on financial resources and employment situation; May – July 2021. Model estimates are adjusted for sex, age, number of chronic health conditions, positive COVID-19 test before Q2, vaccination status, size of household (living alone vs living with other persons), median loneliness score, changes in financial resources, changes in workload and language. Change in employment situation is compared to a reference category of “full- or part-time employed at both Q1 and Q2”

In multivariable analyses (Fig. 4), we found higher scores in stress and anxiety for those shifting from full to part-time employment. Being in other employment positions both at Q1 and Q2 was associated with higher scores in all outcomes. Coefficients and confidence intervals of the multivariable model are reported in table S6 (Additional file 1: Table S6).

### Interactions

**Fig. 5:**
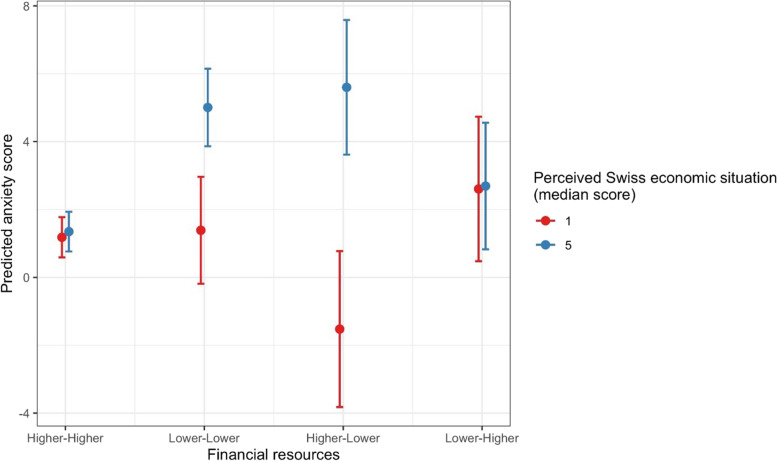
Predicted DASS-21 anxiety scores: marginal effect of financial resources and perceived Swiss economic situation. Note: Higher-Higher = participants had sufficient or comfortable resources at both measurements (Q1 and Q2); Lower-Lower = participants had precarious or insufficient resources at both measurements (Q1 and Q2); Higher-Lower = participants had sufficient or comfortable resources at Q1 and precarious or insufficient at Q2; Lower-Higher = participants had precarious or insufficient at Q1 and sufficient or comfortable at Q2

Perceiving the Swiss economic situation as more worrisome was associated with higher anxiety scores in persons who lost financial resources (comfortable or sufficient at Q1 and became precarious or insufficient at Q2) or had precarious or insufficient resources both at Q1 and Q2. The predicted marginal effects of changes in financial resources and perceived Swiss economic situation are shown in Fig. 5. We also found that a higher perceived risk of getting infected was associated with lower anxiety scores in persons having consistently precarious or insufficient results (Additional File 1: Figure S4). When testing the interaction between changes in employment status and perceived risk of getting infected or perceived Swiss economic situation, we found no results with more accurate predictions [Fig. 5].

### Sensitivity analyses

The results of the sensitivity analyses were consistent with the main analyses and are shown in supplementary material (Additional file 1: Tables S7-10; Figure S5-S6). When running the multivariable linear model using multiple imputation by chained equation, the association between a worsened financial situation and higher anxiety scores disappeared as well as the interaction effect of perceived risk of getting infected on anxiety levels in people with consistently precarious or insufficient results (Additional file 1: Table S7 and Table S10).

## Discussion

In this study, we investigated the impact of changes in financial resources and employment situation on depression, anxiety and stress levels of the general population during and after the second wave of the COVID-19 pandemic in Switzerland. We found that having consistently precarious or insufficient financial resources was associated with poorer mental health outcomes and that an improvement in participants’ financial situation was associated with higher anxiety scores. Moreover, in the working-age population, we found higher scores for stress and anxiety in participants shifting from full to part-time employment. We also found that perceiving the Swiss economic situation as worse was associated with higher anxiety scores in participants with low or decreasing financial resources.

Scores above the DASS-21 thresholds for normal levels were reported by 16% of participants for depression, 8% for anxiety, and 10% for stress. Several systematic reviews have assessed the prevalence of mental health symptoms in the global general population during the pandemic, although estimates vary depending on the country and measurement instruments. In a meta-review including 18 meta-analyses up to March 2021, De Sousa et al. [34] found a prevalence of depression, anxiety and stress in the general population of 27%, 28% and 36%, respectively. In our sample, we found lower estimates, in line with other Swiss studies conducted during the same period [35]. These results could be due to the fact that Switzerland implemented lighter mitigation strategies [2] and had better economic conditions (e.g., lower unemployment rates) compared to many countries included in this review. Moreover, the majority of the studies in the meta-review were performed in Asian countries or included population subgroups that usually have higher prevalence of depressive, stress and anxiety symptoms (e.g., healthcare workers), making a comparison with our data difficult. The proportion of participants who reported depressive symptoms in our study was similar to other assessments conducted earlier (November 2020) in Switzerland that showed a constantly increasing trend in self-reported depressive symptoms during the early pandemic up to November 2020 [36]. Possibly, the psychological resilience after the first phase of the COVID-19 pandemic [37] or the secondary benefits of vaccination (starting at the end of December 2020 in Switzerland), that has been shown to be associated with a reduction in distress [38], could contribute to overall improving mental health outcomes.

Our results are concordant with the evidence that financial insecurity is a risk factor for lower mental health and wellbeing [8]. Other studies have reported the negative impact of these stressors on mental health during the pandemic [39, 40], with possible detrimental consequences (e.g., child maltreatment, domestic violence, substance abuse), especially among vulnerable population groups [41]. In our study we also found an association between an increase of financial resources and higher anxiety scores. While this finding may appear counterintuitive, a possible explanation could be that people who improved their financial situation during the pandemic experienced changes in their work conditions that could be associated with higher anxiety, such as increased job demands and responsibilities. Moreover, an increase in financial resources could also indicate a situation, such as self-employment, which is usually associated with varying levels of income throughout the year and can be more destabilizing than a stable income.

Regarding the effect of perceived economic and infection risks, we found that participants who perceived a higher risk of getting infected had higher stress scores and those who perceived a worse economic situation had slightly higher anxiety, stress and depression scores. Various studies described the impact of the perceived risk of getting infected with COVID-19 on mental health [23, 42]; for instance, Terraneo et al. found a positive association between risk perception and reported depression in six European countries. However, despite the potential negative effects of a higher perceived risk of getting infected on mental health, this could likely be a key motivator for protective behaviours [43], and the balance between the positive and negative effects is difficult to assess. Additionally, our findings showed that perceiving the economic situation as more worrisome modified the association between the loss of financial resources and anxiety scores, increasing anxiety symptoms in an already vulnerable population group. This result is in line with the social amplification of risk framework [44].

This study has some limitations. We had no information regarding participants’ history of mental health symptoms before the pandemic and, therefore, we could not assess pre/post pandemic and further longitudinal changes. We cannot infer causal effects, since we could not rule out the role of unmeasured variables with potential confounding (e.g., low social support network). Further, although a random representative sample was invited to the study, selection effects could not be excluded, such as higher participation of persons with better mental health status or a higher socioeconomic status. Additionally, Q1 and Q2 assessments were completed approximately half a year apart for most of the participants, with only a limited number of participants changing their socioeconomic situation, and the questions on financial situation referred to the previous 6 months, therefore the periods reported in Q1 and Q2 might overlap in some cases. Moreover, although our sample was sufficiently large for the main analyses, it limited some sensitivity analyses (e.g., with dichotomous outcome or in the not retired subgroup of participants). Thus, although sensitivity analyses matched the results of the main analyses in terms of consistent effect estimates, wider confidence intervals meant that we could not reproduce the statistical significance of all results. Finally, we lacked detailed information on participants’ specific job sectors, which would have improved interpretability of the results on the changes in employment situation. Strengths of this study include the assessment of mental health with a previously validated tool, and the fact that changes in financial resources and employment situation were longitudinally assessed before mental health assessments. Moreover, the population-based design of this study improves the generalisability of our results for overall Swiss population.

## Conclusion

This study confirms the negative association between economic constraints and mental health and adds to a growing literature on social determinants of mental health during the COVID-19 pandemic. Further, it offers an insight into the relationship between risk perception or perception of the economic situation and mental health. Public authorities and the media should be aware of the role that people's perceptions can play on public mental health.

## Supplementary Information

**Additional file 1.** Supplementary figures and tables

## Data Availability

Deidentified individual participant data underlying the findings of this study will be available for researchers submitting a methodologically sound proposal to achieve the aims of the proposal after the publication of this article. Access to data requires contacting Corona Immunitas.

## Abbreviations

COVID-19: Coronavirus disease 2019
SARS-CoV-2: Severe acute respiratory syndrome coronavirus 2
CI-DFU eCohort: Corona Immunitas digital follow-up eCohort
DASS-21: Depression, Anxiety and Stress Scale
PCR: Polymerase chain reaction
Q1: First administration of the questionnaire on financial resources and employment situation
Q2: Second administration of the questionnaire on financial resources and employment situation
IQR: Interquartile range
CI: Confidence interval
SSPH +: Swiss School of Public Health
