# Supplementary material for: Changes in socioeconomic resources and mental health after the second COVID-19 wave (2020–2021): a longitudinal study in Switzerland
Source: Int J Equity Health. 2023 Mar 23;22:51. doi: 10.1186/s12939-023-01853-2 (PMC10035489; doi:10.1186/s12939-023-01853-2)
Supplement: Supplementary file 1 — Additional file 1. Supplementary figures and tables [file 12939_2023_1853_MOESM1_ESM.docx]

**Supplementary material**

**“Changes in socioeconomic resources and mental health after the second COVID-19 wave (2020-2021): a longitudinal study in Switzerland”**

**Contents**

**[Figure S1.](#_Toc128734342)** [Timeline of the study and COVID-19 pandemic context 2](#_Toc128734342)

[**Figure S2.** Flow chart of study participants 2](#_Toc128734343)

[**Figure S3.** Theoretical framework of the study 3](#_Toc128734344)

[**Figure S4**: Predicted DASS-21 anxiety scores: marginal effect of financial resources and perceived risk of getting infected. 3](#_Toc128734345)

[**Figure S5**: Multivariate logistic regression model; Predicted probability of moderate to severe DASS-21 anxiety scores: marginal effect of financial resources and perceived Swiss economic situation 4](#_Toc128734346)

[**Figure S6.** Changes in financial resources and mental health outcomes: multivariable linear regression model. Sensitivity analysis with monthly instead of median values of risk perceptions. 5](#_Toc128734347)

[**Table S1.** Comparison of characteristics of the included and excluded population 6](#_Toc128734348)

[**Table S2.** Comparison of financial resources between the included and excluded population 8](#_Toc128734349)

[**Table S3.** DASS-21 severity categories 8](#_Toc128734350)

[**Table S4.** Changes in financial resources and employment situation 9](#_Toc128734351)

[**Table S5.** Changes in financial resources, perceived risk of infection and economic perceptions, and mental health outcomes: multivariable linear regression model. 10](#_Toc128734352)

[**Table S6.** Changes in employment situation and mental health outcomes among working-age participants: multivariable linear regression 11](#_Toc128734353)

[**Table S7.** Changes in financial resources, perceived risk of infection and economic perceptions, and mental health outcomes: multivariable linear regression model. Sensitivity analysis using multiple imputation by chained equations. 12](#_Toc128734354)

[**Table S8.** Changes in employment situation and mental health outcomes among working-age participants: multivariable linear regression. Sensitivity analysis using Multiple imputation by chained equations. 13](#_Toc128734355)

[**Table S9.** Interaction effect of perception of the Swiss economic situation on the association between changes in financial resources and DASS-21 anxiety scores. Sensitivity analysis using Multiple imputation by chained equations. 14](#_Toc128734356)

[**Table S10.** Changes in financial resources, perceived risk of infection and economic perceptions, and mental health outcomes: multivariable logistic regression model. 14](#_Toc128734357)

# **Figure S1.** Timeline of the study and COVID-19 pandemic context


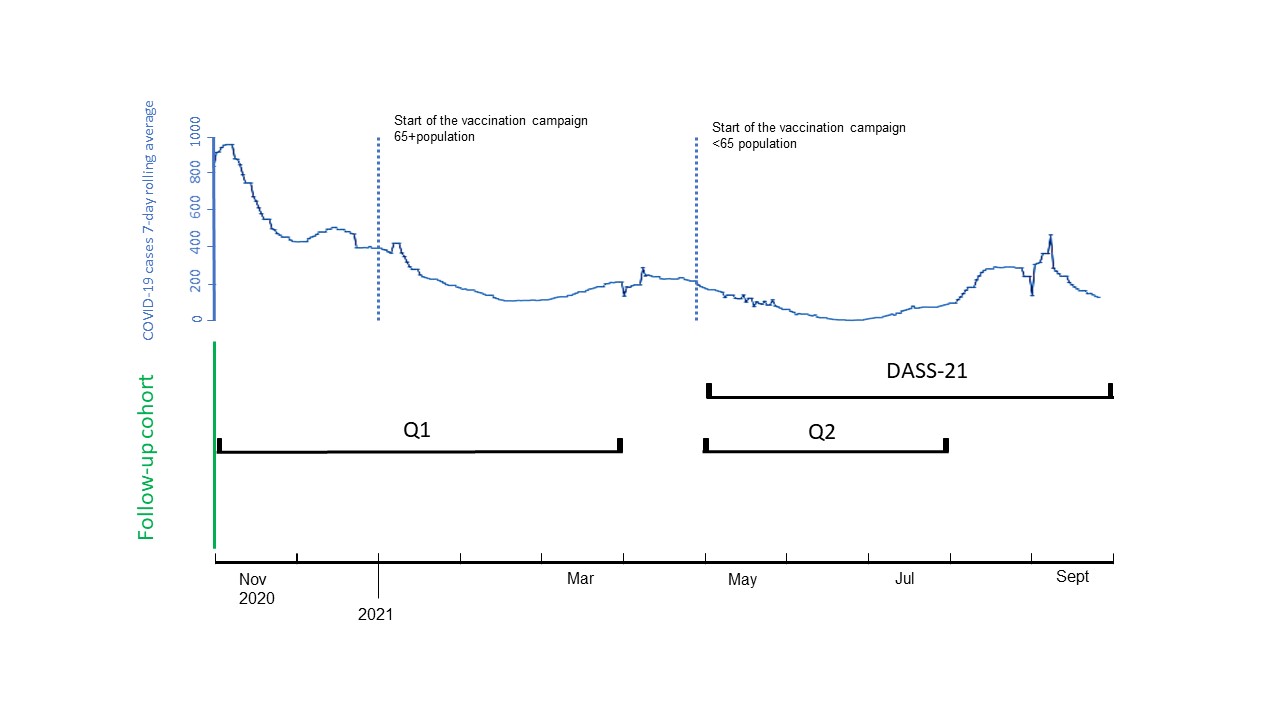


Note: 79% (1385/1759) of the included participants filled the Q1 questionnaire between November and December 2020; 99% (1738/1759) filled the Q2 questionnaire in May 2021; and 98% (1719/1759) of participants filled the DASS-21 questionnaire between June and July 2021

**Figure S2.** Flow chart of study participants**
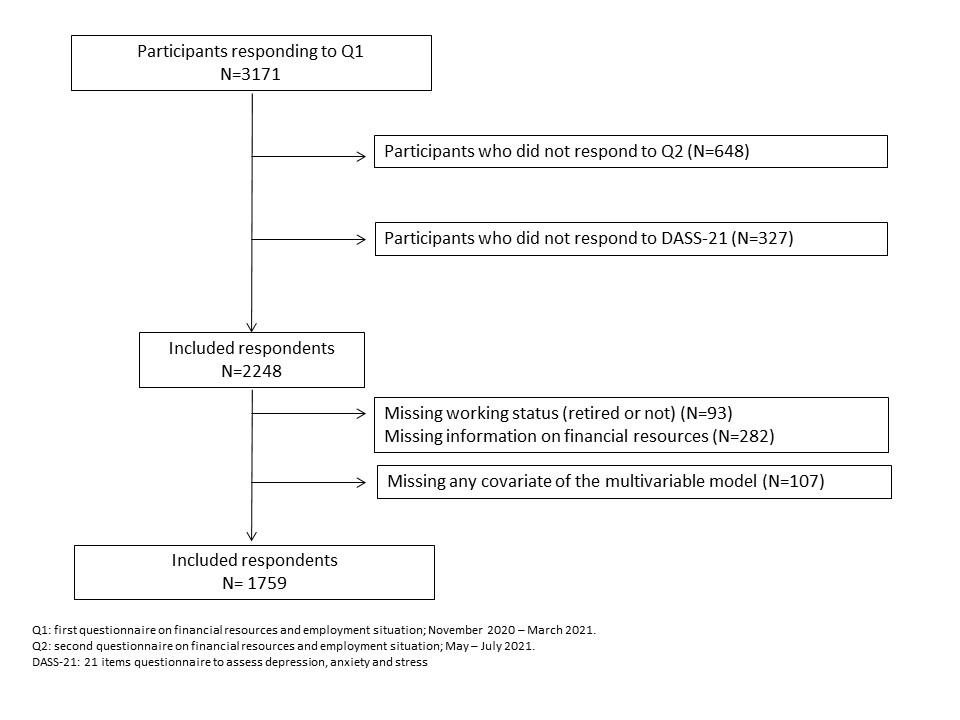
**

# **Figure S3.** Theoretical framework of the study

**
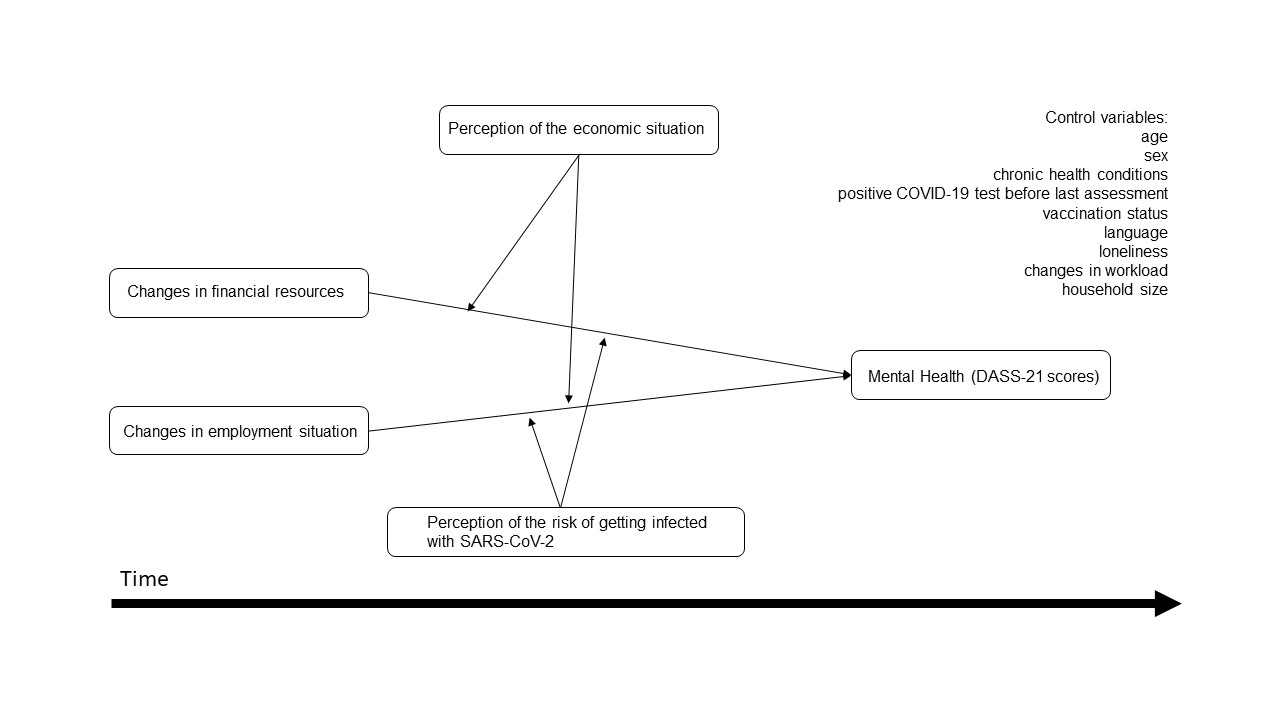
**

# **Figure S4**: Predicted DASS-21 anxiety scores: marginal effect of financial resources and perceived risk of getting infected.


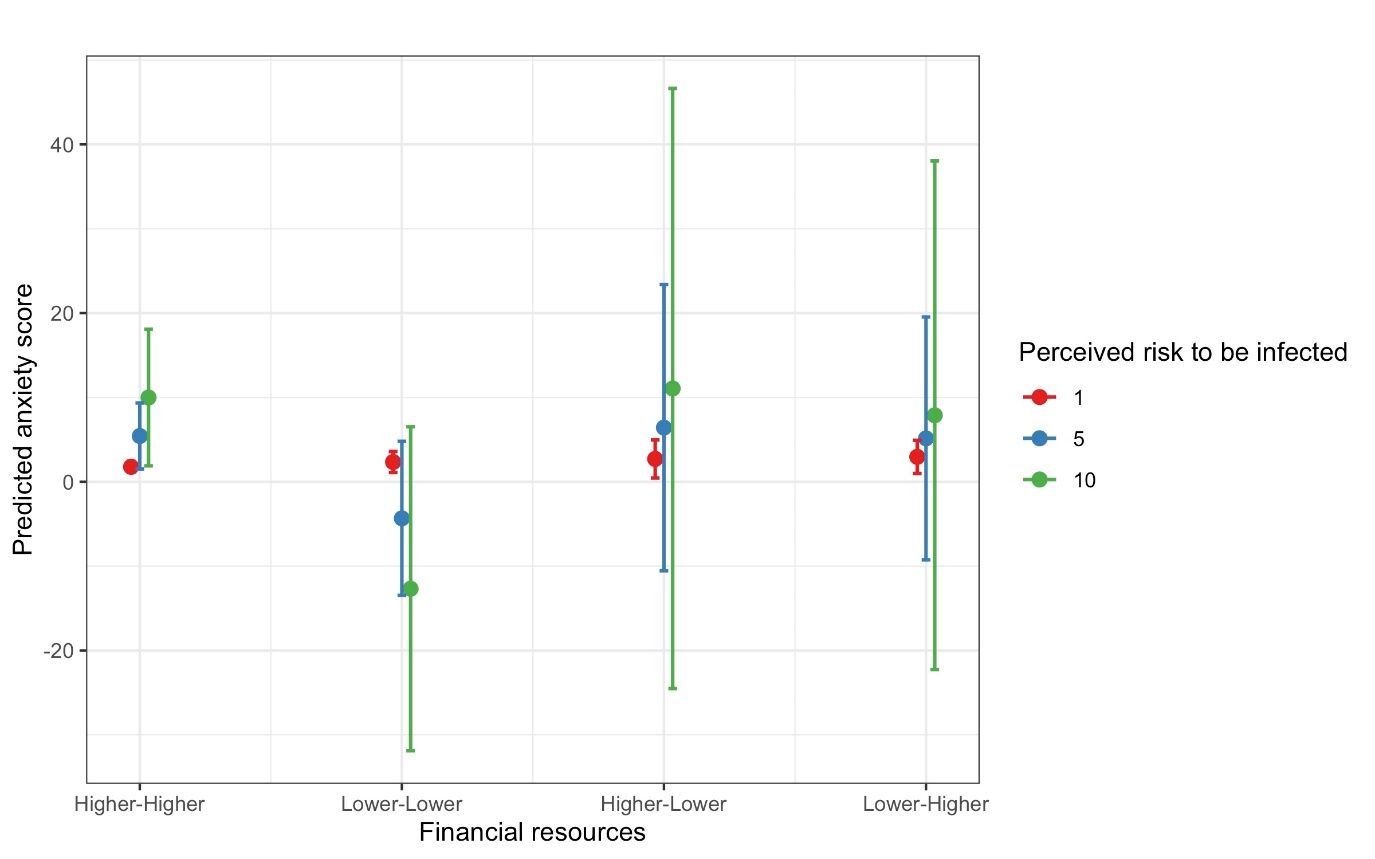


Note: Higher-Higher= participants had sufficient or comfortable resources at both measurements (Q1 and Q2); Lower-Lower= participants had precarious or insufficient resources at both measurements (Q1 and Q2); Higher-Lower= participants had sufficient or comfortable resources at Q1 and precarious or insufficient at Q2; Lower-Higher= participants had precarious or insufficient at Q1 and sufficient or comfortable at Q2.

# **Figure S5**: Multivariate logistic regression model; Predicted probability of moderate to severe DASS-21 anxiety scores: marginal effect of financial resources and perceived Swiss economic situation


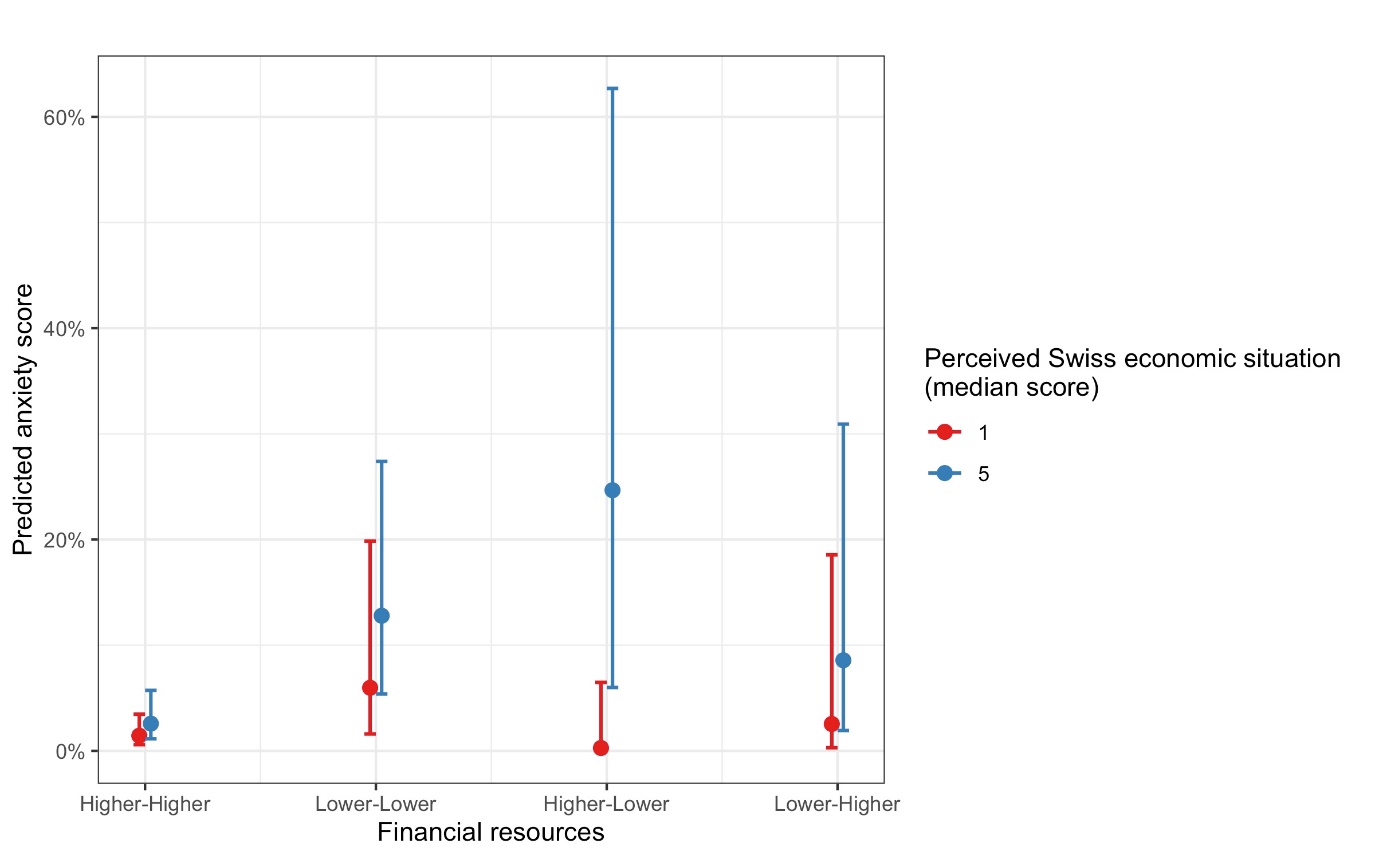


Note: Higher-Higher= participants had sufficient or comfortable resources at both measurements (Q1 and Q2); Lower-Lower= participants had precarious or insufficient resources at both measurements (Q1 and Q2); Higher-Lower= participants had sufficient or comfortable resources at Q1 and precarious or insufficient at Q2; Lower-Higher= participants had precarious or insufficient at Q1 and sufficient or comfortable at Q2.

# **Figure S6.** Changes in financial resources and mental health outcomes: multivariable linear regression model. Sensitivity analysis with monthly instead of median values of risk perceptions.


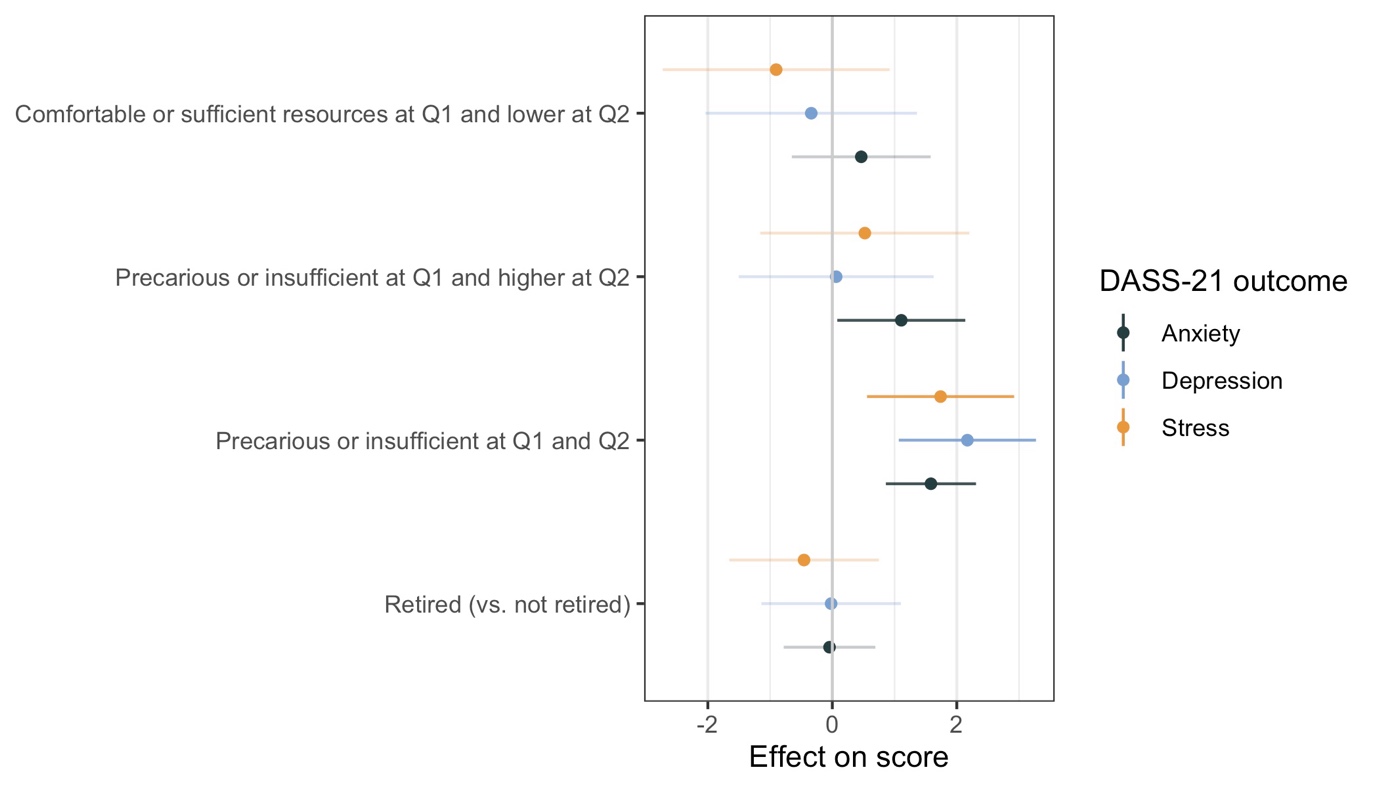


Note: a positive coefficient (effect on score) means a higher DASS-21 score (more symptoms)

Q1: first questionnaire on financial resources and employment situation; November 2020 – March 2021.
Q2: second questionnaire on financial resources and employment situation; May – July 2021.

Model estimates are adjusted for sex, age, number of chronic health conditions, positive COVID-19 test before Q2, vaccination status, size of household (living alone vs living with other persons), median loneliness score, employment status, changes in workload and language.

Financial resources categories are compared to the reference category of “comfortable or sufficient resources at both Q1 and Q2”.

# **Table S1.** Comparison of characteristics of the included and excluded population

|  | **Included** | **Excluded** | **p-value** |
| --- | --- | --- | --- |
| **N** | 1759 | 437 |  |
| **Age,** median (IQR) | 53 (40 - 64) | 56 (41, 66) | 0.2 |
| **Sex** |  |  | <0.001 |
| Female | 909 (52%) | 280 (65%) |  |
| Male | 846 (48%) | 153 (35%) |  |
| Other | 4(0.1%) | 0 (0%) |  |
| Missings | 0 | 4 |  |
| **One or more chronic health conditions** | 621 (35%) | 158 (36%) | 0.7 |
| Missings | 0 | 3 |  |
| **SARS-CoV-2 positive test** | 154 (8.8%) | 24 (5.5%) | 0.025 |
| **SARS-CoV-2 Vaccinated** | 397 (23%) | 117 (27%) | 0.063 |
| **Language** |  |  | 0.5 |
| German | 886 (50%) | 222 (51%) |  |
| Italian | 505 (29%) | 128 (29%) |  |
| French | 353 (20%) | 81 (19%) |  |
| English | 15 (0.9%) | 6 (1.4%) |  |
| **Living alone** | 278 (16%) | 67 (16%) | 0.9 |
| Missings | 0 | 21 |  |
| **Loneliness score,** median (IQR)^b^ | 5 (4 - 7) | 6 (4 - 8) | 0.038 |
| **Worries about Swiss economy,** median (IQR)^b^ | 3 (2 - 4) | 3 (2 - 4) |  |
| **Perceived risk to be infected,** median (IQR)^b^ | 4.4 (2.1 - 6.5) | 4.6 (2.3 - 6.5) | 0.5 |
| Missings | 0 | 71 |  |
| **Change in workload** |  |  | 0.3 |
| No change | 1,046 (78%) | 278 (82%) |  |
| Increase | 150 (11%) | 30 (8.8%) |  |
| Decrease | 140 (10%) | 31 (9.1%) |  |
| Missings | 423^a^ | 98 |  |
| **Mental health outcomes**^c^ |  |  |  |
| **Depression** |  |  |  |
| Median (IQR) | 0 (0 - 6) | 0 (0 - 4) | 0.6 |
| Categories: |  |  | 0.3 |
| Normal | 1,472 (84%) | 367 (84%) |  |
| Mild to moderate | 227 (13%) | 49 (11%) |  |
| Sever to extremely severe | 60 (3.4%) | 21 (4.8%) |  |
| **Anxiety** |  |  |  |
| Median (IQR) | 0 (0 - 2) | 0 (0 - 2) | 0.6 |
| Categories: |  |  | 0.3 |
| Normal | 1,613 (92%) | 400 (92%) |  |
| Mild to moderate | 107 (6.1%) | 22 (5.0%) |  |
| Sever to extremely severe | 39 (2.2%) | 15 (3.4%) |  |
| **Stress** |  |  |  |
| Median (IQR) | 2 (0 - 10) | 2 (0 - 10) | 0.7 |
| Categories |  |  | 0.11 |
| Normal | 1,581 (90%) | 394 (90%) |  |
| Mild to moderate | 136 (7.7%) | 26 (5.9%) |  |
| Sever to extremely severe | 42 (2.4%) | 17 (3.9%) |  |

Note: p-values were computed using Fisher's exact test; Wilcoxon rank sum test; Pearson's Chi-squared test.

# **Table S2.** Comparison of financial resources between the included and excluded population

|  | **Included (1759)** | **Excluded (437)** | **P values** |
| --- | --- | --- | --- |
| **Financial resources Q1** |  |  | 0.003 |
| Comfortable | 175 (43%) | 93 (34%) |  |
| Sufficient | 191 (47%) | 119 (43%) |  |
| Precarious | 37 (9.2%) | 58 (21%) |  |
| Insufficient | 29 (1.6%) | 4 (1.5%) |  |
| Missings | 0 | 163 |  |
| **Financial resources Q2** |  |  | 0.001 |
| Comfortable | 783 (45%) | 93 (39%) |  |
| Sufficient | 724 (41%) | 87 (37%) |  |
| Precarious | 219 (12%) | 51 (22%) |  |
| Insufficient | 33 (1.9%) | 6 (2.5%) |  |
| Missings | 0 | 200 |  |

Note: p-values were computed using Pearson's Chi-squared test.

# **Table S3.** DASS-21 severity categories

| Severity | Depression | Anxiety | Stress |
| --- | --- | --- | --- |
| Normal | 0-9 | 0-7 | 0-14 |
| Mild | 10-13 | 8-9 | 15-18 |
| Moderate | 14-20 | 10-14 | 19-25 |
| Severe | 21-27 | 15-19 | 26-33 |
| Extremely severe | 28+ | 20+ | 34+ |

Note: each category score is calculated by summing the subscale item scores and multiplying by two.

# **Table S4.** Changes in financial resources and employment situation

Changes in financial resources: all participants included in the models

|  | Comfortable/sufficient at Q2 | Precarious/insufficient at Q2 |
| --- | --- | --- |
| Comfortable/sufficient at Q1 | 1421 | 68 |
| Precarious/insufficient at Q1 | 86 | 184 |

Changes in financial resources: working population

|  | Comfortable/sufficient at Q2 | Precarious/insufficient at Q2 |
| --- | --- | --- |
| Comfortable/sufficient at Q1 | 1053 | 53 |
| Precarious/insufficient at Q1 | 71 | 159 |

Changes in employment status: working population

|  | Full time employed | Part time employed | Self employed | Retired | Other | Missing Q2 information |
| --- | --- | --- | --- | --- | --- | --- |
| Full time employed | 588 | 32 | 11 | 6 | 13 | 0 |
| Part time employed | 32 | 298 | 5 | 8 | 17 | 1 |
| Self employed | 5 | 7 | 113 | 9 | 6 | 1 |
| Retired | 0 | 0 | 0 | 0 | 0 | 0 |
| Other | 10 | 17 | 2 | 11 | 144 | 0 |

# **Table S5.** Changes in financial resources, perceived risk of infection and economic perceptions, and mental health outcomes: multivariable linear regression model.

Full study sample (n=1759)

|  | Anxiety | Depression | Stress | Adjusted GVIF |
| --- | --- | --- | --- | --- |
|  | β (95%CI) | β (95%CI) | β (95%CI) |  |
| Financial resources |  |  |  | 1.0 |
| Comfortable/sufficient at Q1 and Q2 | [ref] | [ref] | [ref] |  |
| Comfortable/sufficient at Q1 and lower at Q2 | 1.1 (0.18, 2.1) | 0.73 (-0.66, 2.1) | 0.69 (-0.83, 2.2) |  |
| Precarious/insufficient at Q1 and higher at Q2 | 1.3 (0.50, 2.2) | 0.71 (-0.54, 2.0) | 0.89 (-0.47, 2.2) |  |
| Precarious/insufficient at Q1 and Q2 | 2.1 (1.5, 2.7) | 2.5 (1.6, 3.4) | 2.3 (1.3, 3.3) |  |
| Perceived risk to be infected | 0.54 (-0.22, 1.3) | 0.72 (-0.41, 1.8) | 1.8 (0.58, 3.0) | 1.1 |
| Perceived Swiss economic situation | 0.21 (0.01, 0.42) | 0.37 (0.06, 0.67) | 0.40 (0.07, 0.73) | 1.1 |

Non-retired study sample (n=1336)

|  | Anxiety | Depression | Stress | Adjusted GVIF |
| --- | --- | --- | --- | --- |
|  | β (95%CI) | β (95%CI) | β (95%CI) |  |
| Financial resources |  |  |  | 1.0 |
| Comfortable/sufficient at Q1 and Q2 | [ref] | [ref] | [ref] |  |
| Comfortable/sufficient at Q1 and lower at Q2 | 1.3 (0.16, 2.4) | 0.92 (-0.70, 2.5) | 0.85 (-0.94, 2.6) |  |
| Precarious/insufficient at Q1 and higher at Q2 | 1.3 (0.32, 2.3) | 1.0 (-0.47, 2.4) | 1.0 (-0.53, 2.6) |  |
| Precarious/insufficient at Q1 and Q2 | 2.2 (1.5, 2.9) | 2.1 (1.1, 3.1) | 2.3 (1.1, 3.4) |  |
| Perceived risk to be infected | 0.74 (-0.20, 1.7) | 1.1 (-0.25, 2.5) | 2.5 (1.0, 4.0) | 1.1 |
| Perceived Swiss economic situation | 0.25 (0.00, 0.49) | 0.40 (0.05, 0.76) | 0.41 (0.02, 0.80) | 1.1 |

Note: Q1: first questionnaire on financial resources and employment situation; November 2020 – March 2021.
Q2: second questionnaire on financial resources and employment situation; May – July 2021.

Model estimates are adjusted for sex, age, number of chronic health conditions, positive COVID-19 test before Q2, vaccination status, size of household (living alone vs living with other persons), median loneliness score, employment status, changes in workload and language.

GVIF = Generalized Variance Inflation Factor

Adjusted GVID= GVIF^[1/(2*df)]

# **Table S6.** Changes in employment situation and mental health outcomes among working-age participants: multivariable linear regression

|  | Anxiety | Depression | Stress | Adjusted GVIF |
| --- | --- | --- | --- | --- |
| n=1336 | β (95%CI) | β (95%CI) | β (95%CI) | 1.0 |
| Full-time or part-time at both Q1 and Q2 | [ref] | [ref] | [ref] |  |
| Full-time at Q1 and part-time at Q2 | 1.9 (0.49, 3.3) | 1.8 (-0.25, 3.9) | 3.3(1.0, 5.6) |  |
| Part-time at Q1 and full-time at Q2 | -0.05 (-1.5, 1.4) | -1.3 (-3.4, 0.77) | -0.35(-2.6, 1.9) |  |
| Full- or part-time at Q1 and self-employed at Q2 | -1.1 (-3.1, 0.85) | -2.4 (-5.3, 0.45) | -3.0(-6.2, 0.22) |  |
| Self-emplyed at Q1 and full- or part-time at Q2 | -1.6 (-3.9, 0.73) | -0.70 (-4.0, 2.6) | -1.0(-4.7, 2.7) |  |
| Self-emplyed at Q1 and Q2 | 0.03 (-0.79, 0.85) | -0.46 (-1.6, 0.73) | 0.28(-1.0, 1.6) |  |
| Full- or part-time at Q1 and other at Q2 | 0.85 (-0.65, 2.4) | 0.91 (-1.3, 3.1) | 0.20(-2.2, 2.6) |  |
| Other employment at Q1 and Q2 | 0.82 (0.07, 1.6) | 1.7 (0.63, 2.8) | 1.6(0.35, 2.8) |  |
| Other at Q1 and full- or part-time at Q2 | -0.20 (-1.8, 1.4) | 1.0 (-1.3, 3.2) | 1.5(-1.0, 4.0) |  |

Note: Q1: first questionnaire on financial resources and employment situation; November 2020 – March 2021.
Q2: second questionnaire on financial resources and employment situation; May – July 2021.

Model estimates are adjusted for sex, age, number of chronic health conditions, positive COVID-19 test before Q2, vaccination status, size of household (living alone vs living with other persons), median loneliness score, changes in financial resources, changes in workload and language.

GVIF = Generalized Variance Inflation Factor

Adjusted GVID= GVIF^[1/(2*df)]

# **Table S7.** Changes in financial resources, perceived risk of infection and economic perceptions, and mental health outcomes: multivariable linear regression model. Sensitivity analysis using multiple imputation by chained equations.

Full study sample (n=2196)

|  | Anxiety | Depression | Stress |
| --- | --- | --- | --- |
|  | β (95%CI) | β (95%CI) | β (95%CI) |
| Financial resources |  |  |  |
| Comfortable/sufficient at Q1 and Q2 | [ref] | [ref] | [ref] |
| Comfortable/sufficient at Q1 and lower at Q2 | 0.92 (-0.02, 1.9) | 0.44 (-0.93, 1.8) | 0.30 (-1.2, 1.8) |
| Precarious/insufficient at Q1 and higher at Q2 | 1.1 (0.29, 2.0) | 0.84 (-0.38, 2.1) | 0.72 (-0.59, 2.04) |
| Precarious/insufficient at Q1 and Q2 | 2.2 (1.6, 2.8) | 2.7 (1.8, 3.5) | 2.4 (1.4, 3.3) |
| Perceived risk to be infected | 0.60 (-0.20, 1.4) | 0.70 (-0.43, 1.8) | 1.8 (0.58, 3.0) |
| Perceived Swiss economic situation | 0.24 (0.04, 0.45) | 0.37 (0.07, 0.67) | 0.44 (0.11, 0.77) |

Non-retired study sample (n=1582)

|  | Anxiety | Depression | Stress |
| --- | --- | --- | --- |
|  | β (95%CI) | β (95%CI) | β (95%CI) |
| Financial resources |  |  |  |
| Comfortable/sufficient at Q1 and Q2 | [ref] | [ref] | [ref] |
| Comfortable/sufficient at Q1 and lower at Q2 | 0.96 (-0.16, 2.1) | 0.48 (-1.1, 2.1) | 0.28 (-1.5, 2.0) |
| Precarious/insufficient at Q1 and higher at Q2 | 1.0 (0.05, 2.0) | 1.0 (-0.34, 2.4) | 0.78 (-0.75, 2.3) |
| Precarious/insufficient at Q1 and Q2 | 2.3 (1.6, 3.0) | 2.2 (1.2, 3.2) | 2.3 (1.2, 3.4) |
| Perceived risk to be infected | 0.85 (-0.17, 1.9) | 1.1 (-0.29, 2.5) | 2.5 (1.0, 4.0) |
| Perceived Swiss economic situation | 0.28 (0.03, 0.52) | 0.40 (0.05, 0.76) | 0.45 (0.06, 0.83) |

Note: Q1: first questionnaire on financial resources and employment situation; November 2020 – March 2021.
Q2: second questionnaire on financial resources and employment situation; May – July 2021.

Model estimates are adjusted for sex, age, number of chronic health conditions, positive COVID-19 test before Q2, vaccination status, size of household (living alone vs living with other persons), median loneliness score, employment status, changes in workload and language.

# **Table S8.** Changes in employment situation and mental health outcomes among working-age participants: multivariable linear regression. Sensitivity analysis using Multiple imputation by chained equations.

|  | Anxiety | Depression | Stress |
| --- | --- | --- | --- |
| n=1582 | β (95%CI) | β (95%CI) | β (95%CI) |
| Full-time or part-time at both Q1 and Q2 | [ref] | [ref] | [ref] |
| Full-time at Q1 and part-time at Q2 | 1.5 (0.1, 2.9) | 1.4 (-0.54, 3.4) | 2.5 (0.30, 4.6) |
| Part-time at Q1 and full-time at Q2 | -0.06 (-1.5, 1.4) | -1.3 (-3.4, 0.72) | -0.18(-2.4, 2.1) |
| Full- or part-time at Q1 and self-employed at Q2 | -1.1 (-3.1, 0.93) | -2.4 (-5.3, 0.50) | -2.9(-6.1, 0.28) |
| Self-emplyed at Q1 and full- or part-time at Q2 | -1.5 (-3.7, 0.80) | -0.55 (-3.8, 2.7) | -0.62(-4.2, 2.9) |
| Self-emplyed at Q1 and Q2 | 0.05 (-0.78, 0.87) | -0.40 (-1.6, 0.77) | 0.35(-0.94, 1.6) |
| Full- or part-time at Q1 and other at Q2 | 0.53 (-0.91, 2.0) | 0.94 (-1.1, 3.0) | -0.03(-2.3, 2.2) |
| Other employment at Q1 and Q2 | 1.2 (0.43, 1.9) | 2.2 (1.1, 3.3) | 1.9(0.67, 3.0) |
| Other at Q1 and full- or part-time at Q2 | -0.24 (-1.8, 1.4) | 1.0 (-1.3, 3.3) | 1.5(-1.0, 4.0) |

Note: Q1: first questionnaire on financial resources and employment situation; November 2020 – March 2021.
Q2: second questionnaire on financial resources and employment situation; May – July 2021.

Model estimates are adjusted for sex, age, number of chronic health conditions, positive COVID-19 test before Q2, vaccination status, size of household (living alone vs living with other persons), median loneliness score, changes in financial resources, changes in workload and language.

# **Table S9.** Interaction effect of perception of the Swiss economic situation on the association between changes in financial resources and DASS-21 anxiety scores. Sensitivity analysis using Multiple imputation by chained equations.

|  | Anxiety |
| --- | --- |
|  | β (95%CI) |
| Financial resources |  |
| Comfortable/sufficient at Q1 and Q2 | [ref] |
| Comfortable/sufficient at Q1 and lower at Q2 | 1.38 (0.38, 2.37) |
| Precarious/insufficient at Q1 and higher at Q2 | -0.04 (-0.97, 0.88) |
| Precarious/insufficient at Q1 and Q2 | 0.85 (0.22, 1.47) |

# **Table S10.** Changes in financial resources, perceived risk of infection and economic perceptions, and mental health outcomes: multivariable logistic regression model.

Full study sample (n=1759)

|  | Anxiety | Depression | Stress |
| --- | --- | --- | --- |
|  | OR (95%CI) | OR (95%CI) | OR (95%CI) |
| Financial resources |  |  |  |
| Comfortable/sufficient at Q1 and Q2 | [ref] | [ref] | [ref] |
| Comfortable/sufficient at Q1 and lower at Q2 | 2.62 (0.87,6.80) | 1.44 (0.51, 3.57) | 0.70 (0.15, 2.32) |
| Precarious/insufficient at Q1 and higher at Q2 | 2.42 (0.92,5.73) | 1.72 (0.76, 3.64) | 1.21 (0.41, 3.09) |
| Precarious/insufficient at Q1 and Q2 | 4.75 (2.61,8.62) | 2.34 (1.37, 3.96) | 2.01 (1.05, 3.77) |
| Perceived risk to be infected | 1.18 (0.98, 1.70) | 1.33 (0.57, 3.10) | 1.34 (0.48, 3.76) |
| Perceived Swiss economic situation | 1.29 (0.43, 3.26) | 1.17 (0.93, 1.46) | 1.26 (0.96, 1.67) |

OR – odds ratio.
Note: Q1: first questionnaire on financial resources and employment situation; November 2020 – March 2021.
Q2: second questionnaire on financial resources and employment situation; May – July 2021.

Model estimates are adjusted for sex, age, number of chronic health conditions, positive COVID-19 test before Q2, vaccination status, size of household (living alone vs living with other persons), median loneliness score, employment status, changes in workload and language.

Outcome were dichotomized as follow: normal or mild vs moderate or severe or extreme
